# Supplementary material for: Unusual Bicyclo[3.2.1]Octanoid Neolignans from Leaves of Piper crocatum and Their Effect on Pyruvate Dehydrogenase Activity
Source: Plants (Basel). 2021 Sep 7;10(9):1855. doi: 10.3390/plants10091855 (PMC8469962; doi:10.3390/plants10091855)
Supplement: Supplementary file 1 [file plants-10-01855-s001.zip › plants-1365581-supplementary.pdf]

Unusual bicyclo [3.2.1] octanoid neolignans from leaves of *Piper crocatum* and their effect on pyruvate dehydrogenase activity.

Yu Juan Chai [1,†], Younghoon Go [2,†], Hai Qi Zhou [3], HongXu Li [3], Sun Joo Lee [4], Yeo Jin Park [2,5], Wahyu Widowatib [6], Rizal Rizal [7], Young Ho Kim [8], Seo Young Yang [9,*], and Wei Li [2,*]

1 School of Biomedical Engineering, Health Science Center, Shenzhen University, Shenzhen 518060, China; chaiyj@szu.edu.cn

2 Korean Medicine (KM) Application Center, Korea Institute of Oriental Medicine, Daegu 41062, Korea; gotra827@kiom.re.kr

3 Shenzhen Key Laboratory of Marine Bioresource and Eco-environmental Science, College of Life Sciences and Oceanography, Shenzhen University, Shenzhen, 518060, China; 2170257307@email.szu.edu.cn

4 New Drug Development Center, Daegu-Gyeongbuk Medical Innovation Foundation, 80 Cheombok-ro, Dong-gu, Daegu 41061, Korea; disjrk@dgmif.re.kr

5 Korean Convergence Medicine, University of Science and Technology, Daejeon 34054, Republic of Korea;

6 Medical Research Centre, Faculty of Medicine, Maranatha Christian University, Bandung, 40164, Indonesia; wahyu_w60@yahoo.com

7 Biomolecular Biomedical Research Center for Biology, Indoensian Institute of Sciences, Bandung, 40163, Indonesia; rizal_biotek@yahoo.com

8 College of Pharmacy, Chungnam National University, Daejeon 34134, Korea; yhk@cnu.ac.kr

9 Department of Pharmaceutical Engineering, Sangji University, 83 Sangjidae-gil, Wonju-si, Gangwon-do, 26339, Republic of Korea

* Correspondence: syyang@sangji.ac.kr (S.Y.Y); liwei1986@kiom.re.kr (W.L)Tel.: +82-42-821-5933 (S.Y.Y.); +82-53-940-3874 (W.L.)

† These authors contributed equally to this work.

Figure S1. ^{1}H-NMR (600 MHz, dimethyl sulfoxide-d_6) spectrum of compound **1**……………………………………………………………4

Figure S2. ^{13}C-NMR (150 MHz, dimethyl sulfoxide-d_6) spectrum of compound **1**…………………………………………………………5

Figure S3. HSQC spectrum of compound **1**…………………………………………………………………………………………6

Figure S4. HMBC spectrum of compound **1**…………………………………………………………………………………………7

Figure S5. COSY spectrum of compound **1**…………………………………………………………………………………………8

Figure S6. ROESY spectrum of compound **1**……………………………………………………………………………………….9

Figure S7. Adjacent torsion angles of compound **1**……………………………………………………………………………10

Figure S7. HR-ESI-MS spectrum of compound **1**…………………………………………………………………………………11

Figure S8. ^{1}H-NMR (600 MHz, acetone-d_6) spectrum of compound **2**…………………………………………………………12

Figure S9. ^{13}C-NMR (150 MHz, acetone-d_6) spectrum of compound **2**………………………………………………………13

Figure S10. HSQC spectrum of compound **2**……………………………………………………………………………………….14

Figure S11. HMBC spectrum of compound **2**…………………………………………………………………………………….15

Figure S12. COSY spectrum of compound **2**………………………………………………………………………………………16

Figure S13. ROESY spectrum of compound **2**…………………………………………………………………………………….17

Figure S14. HR-ESI-MS spectrum of compound **2**………………………………………………………………………………18

Figure S15. ^{1}H-NMR (600 MHz, methanol-d_4) spectrum of compound **3**……………………………………………………19

Figure S16. ^{13}C-NMR (150 MHz, methanol-d_4) spectrum of compound **3**…………………………………………………20

Figure S17. HSQC spectrum of compound **3**……………………………………………………………………………………………21

Figure S18. HMBC spectrum of compound **3**……………………………………………………………………………………………22

Figure S19. COSY spectrum of compound **3**……………………………………………………………………………………………23

Figure S20. ROESY spectrum of compound **3**…………………………………………………………………………………………24

Figure S22. Adjacent torsion angles of compound **3**………………………………………………………………………………25

Figure S23. HR-ESI-MS spectrum of compound **3**…………………………………………………………………………………26

Figure S24. Speculated biosynthetic pathway of compound **1**……………………………………………………………………27

Figure S25. Speculated biosynthetic pathway of compound **3**……………………………………………………………………28

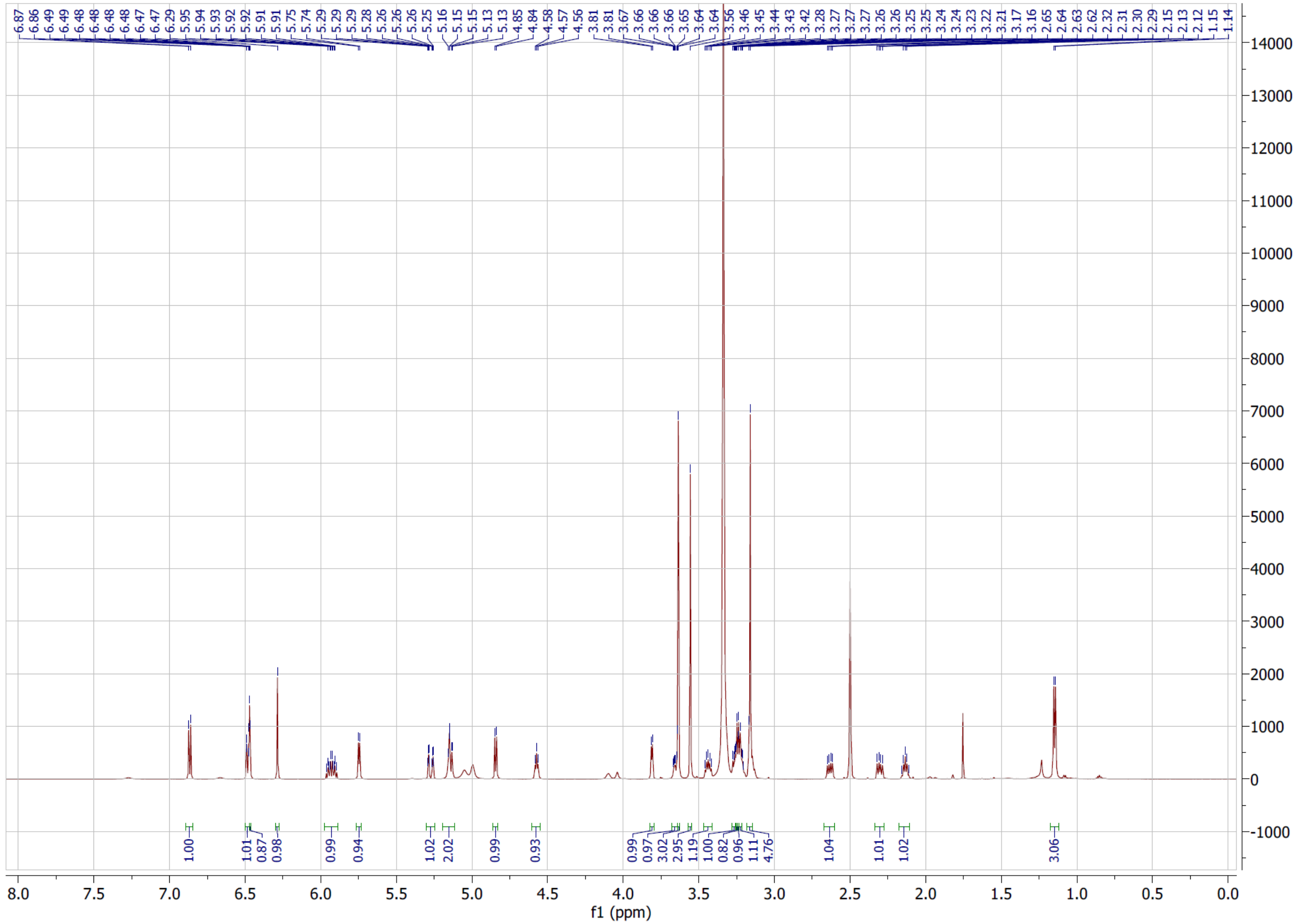

Figure S1. ^{1}H-NMR (600 MHz, dimethyl sulfoxide-d_6) spectrum of compound **1**

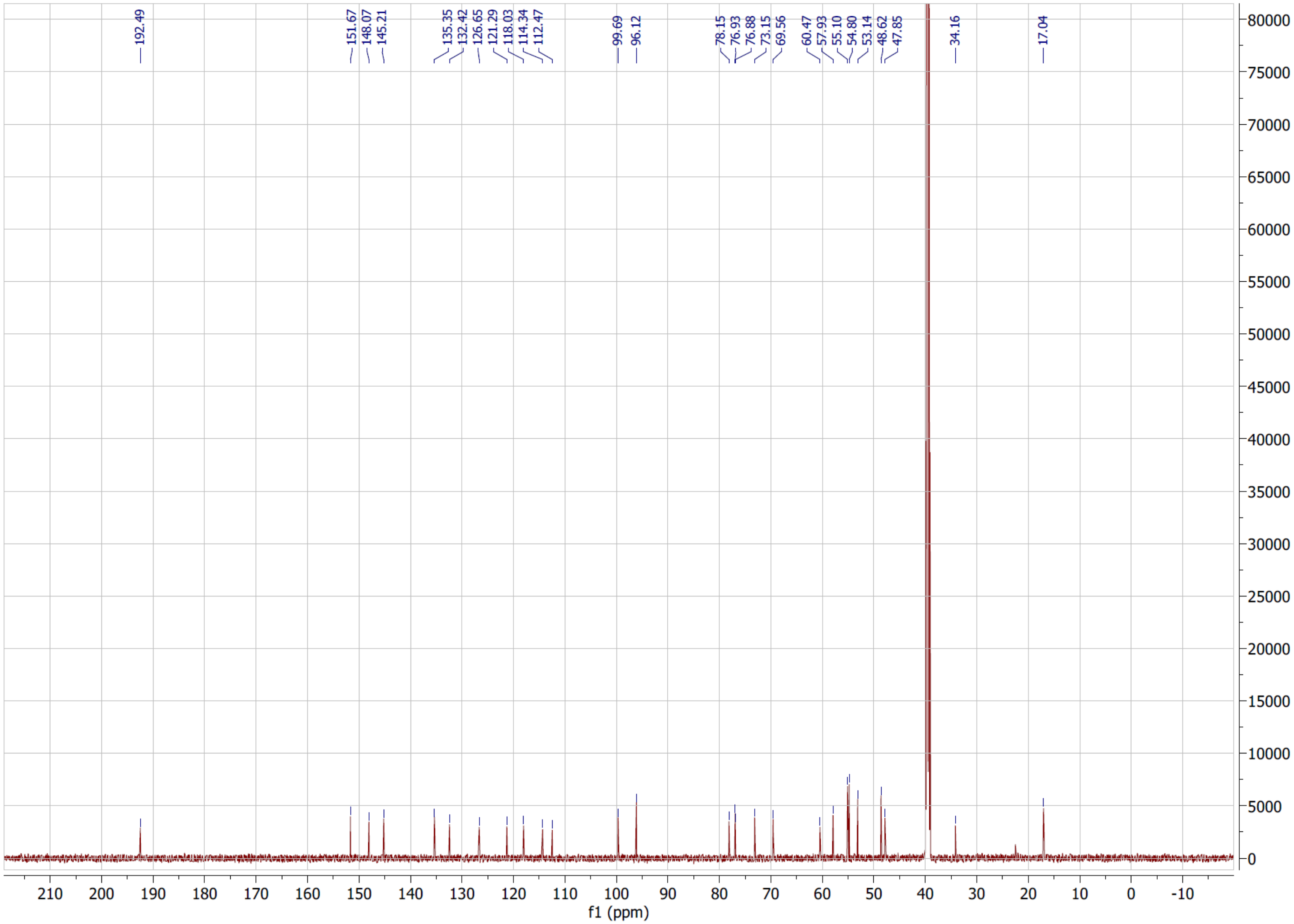

Figure S2. ^{13}C-NMR (150 MHz, dimethyl sulfoxide-d_6) spectrum of compound **1**

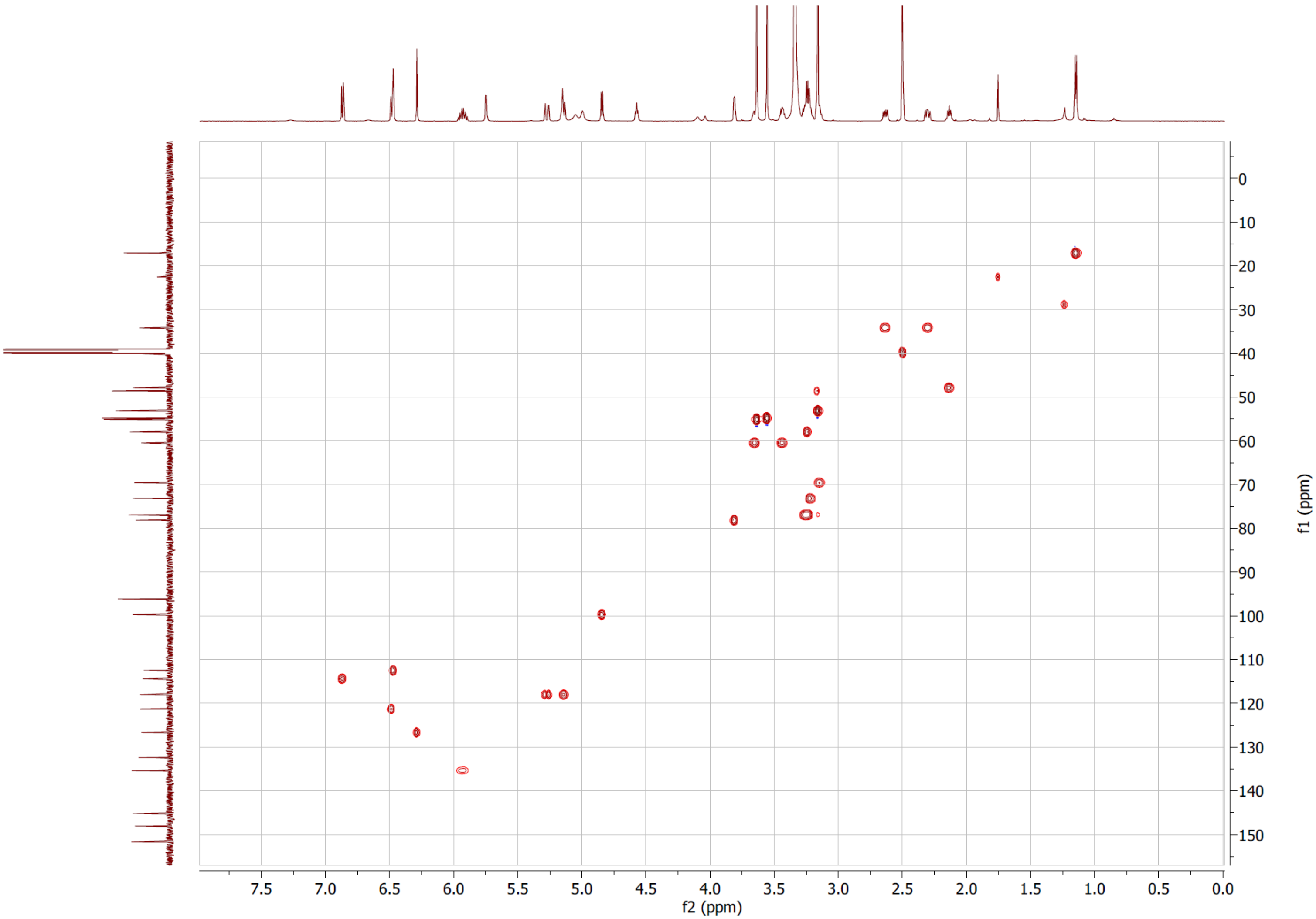

Figure S3. HSQC spectrum of compound **1**

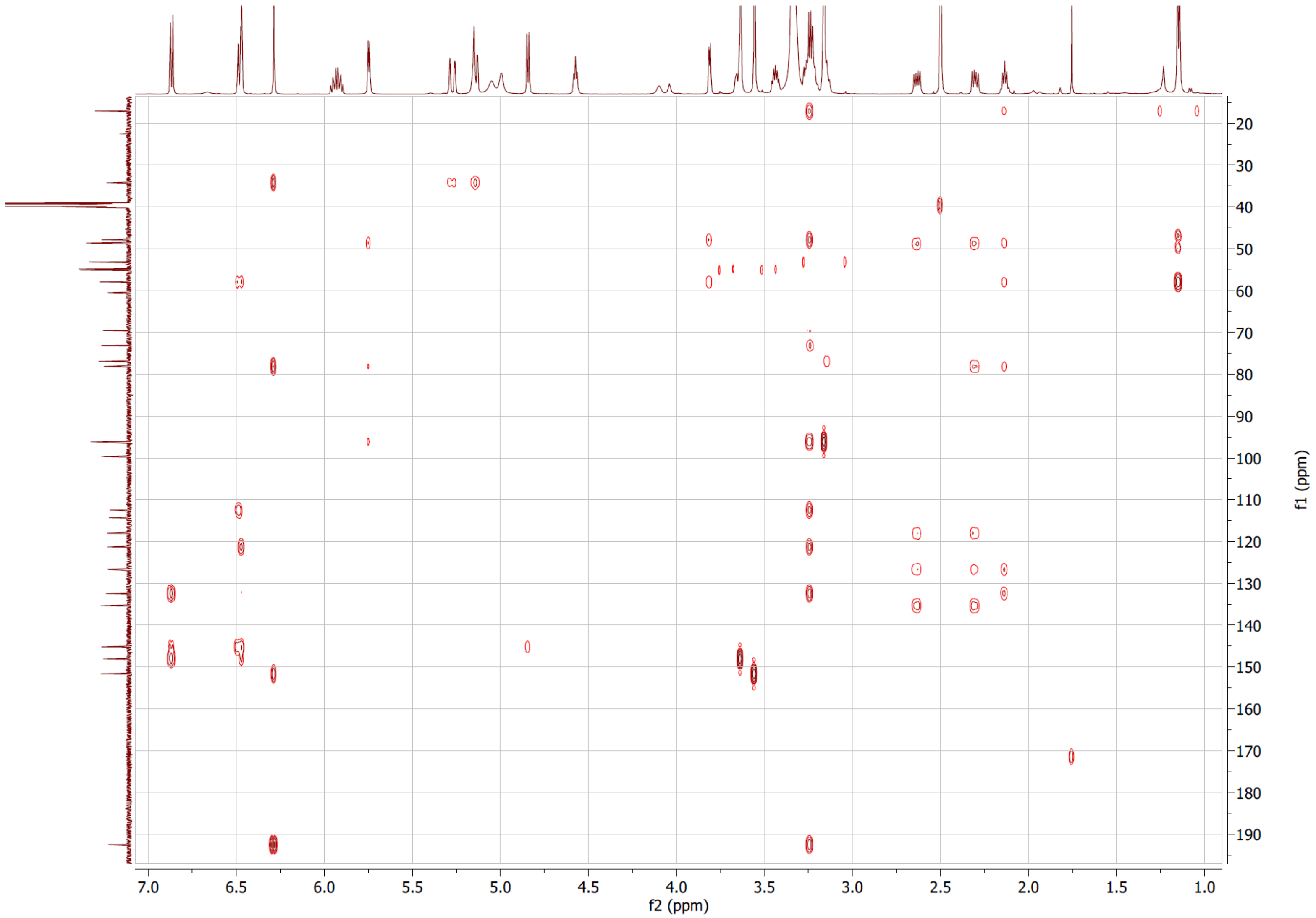

Figure S4. HMBC spectrum of compound **1**

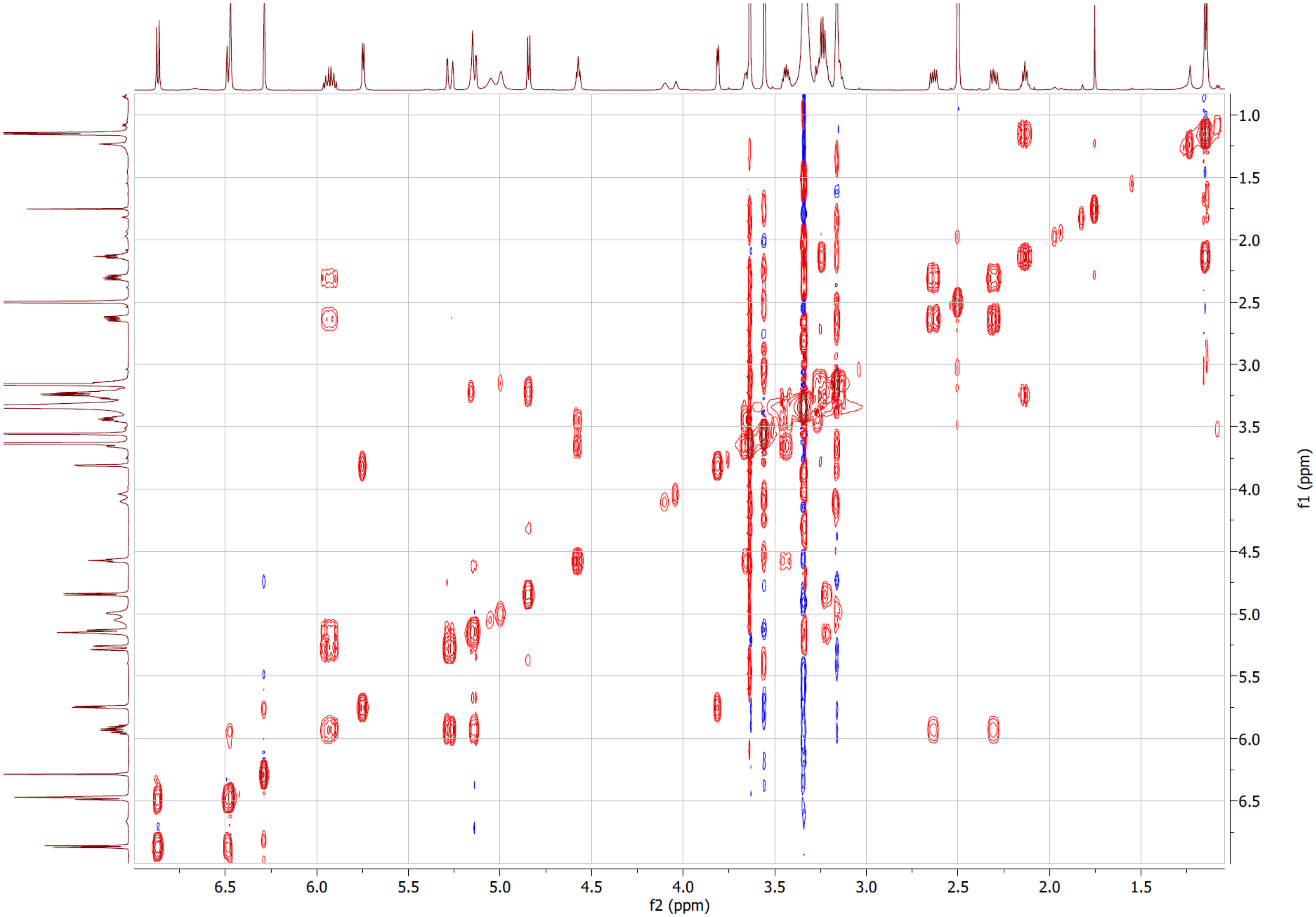

Figure S5. COSY spectrum of compound **1**

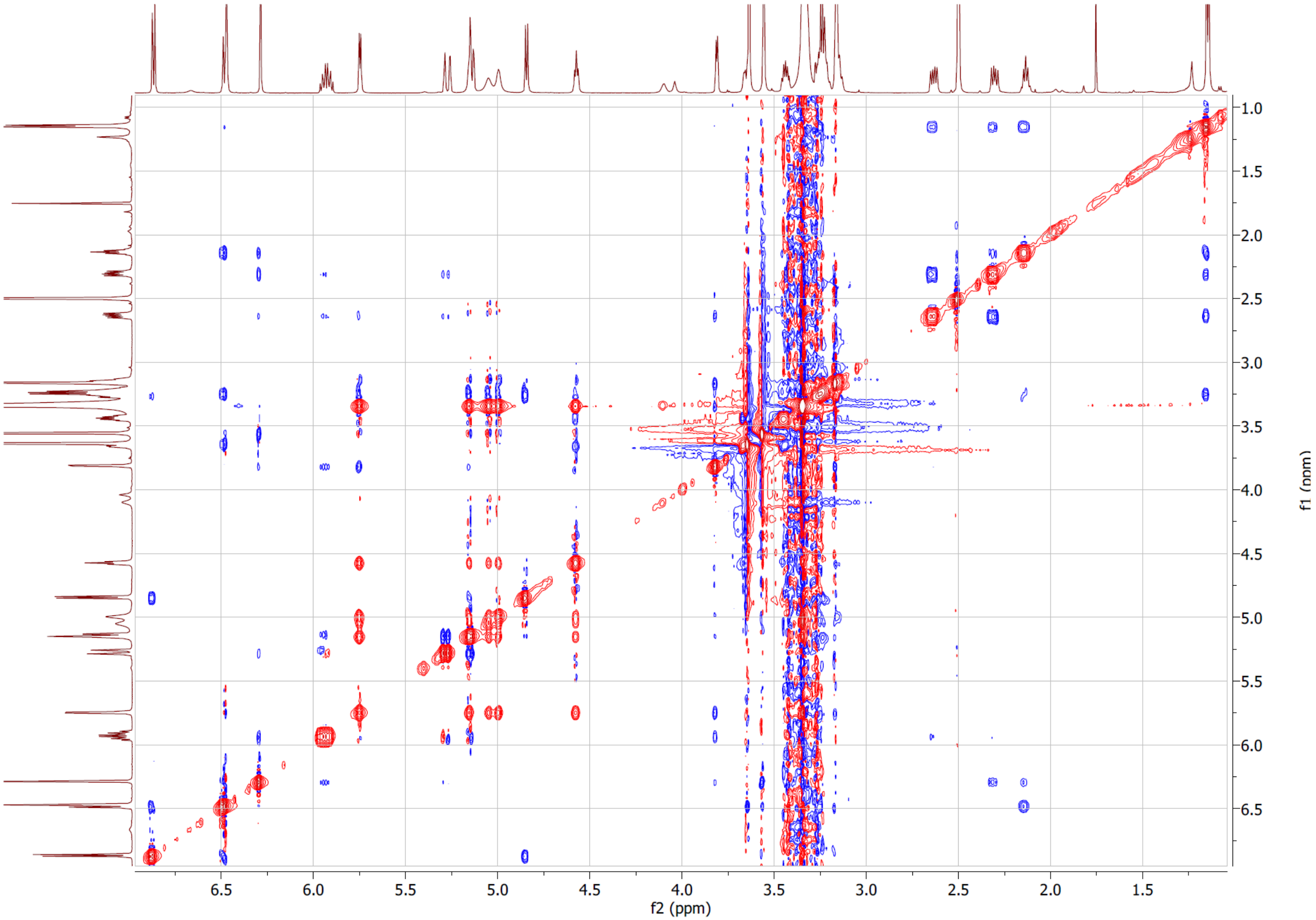

Figure S6. ROESY spectrum of compound

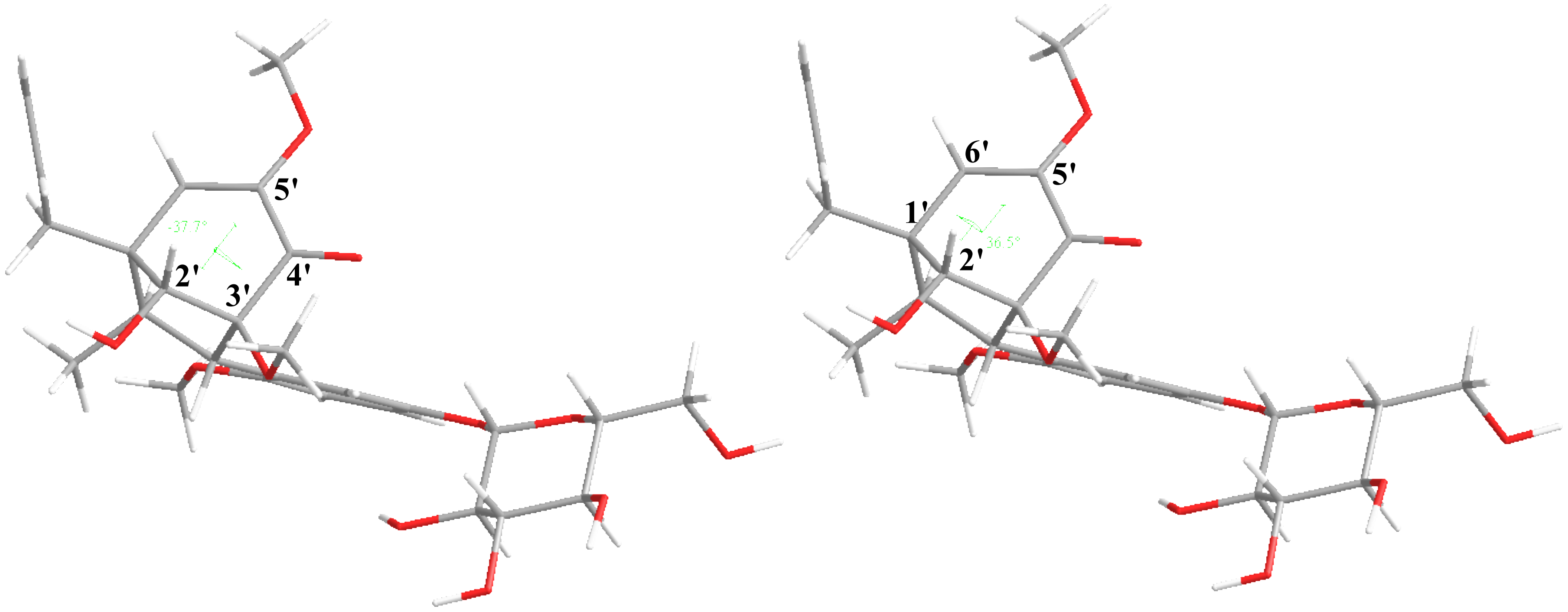

Figure S7. Adjacent torsion angles of compound **1**

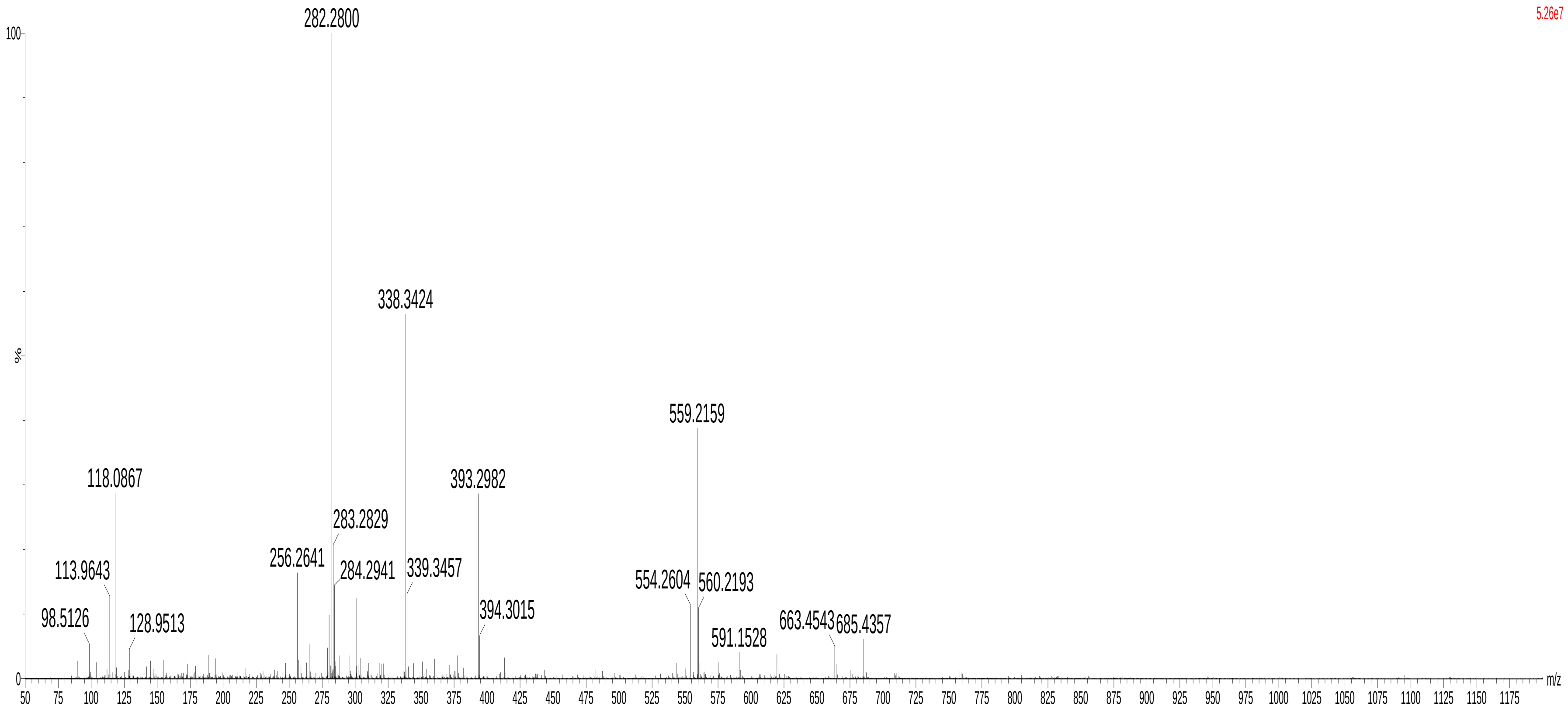

Figure S8. HR-ESI-MS spectrum of compound **1**

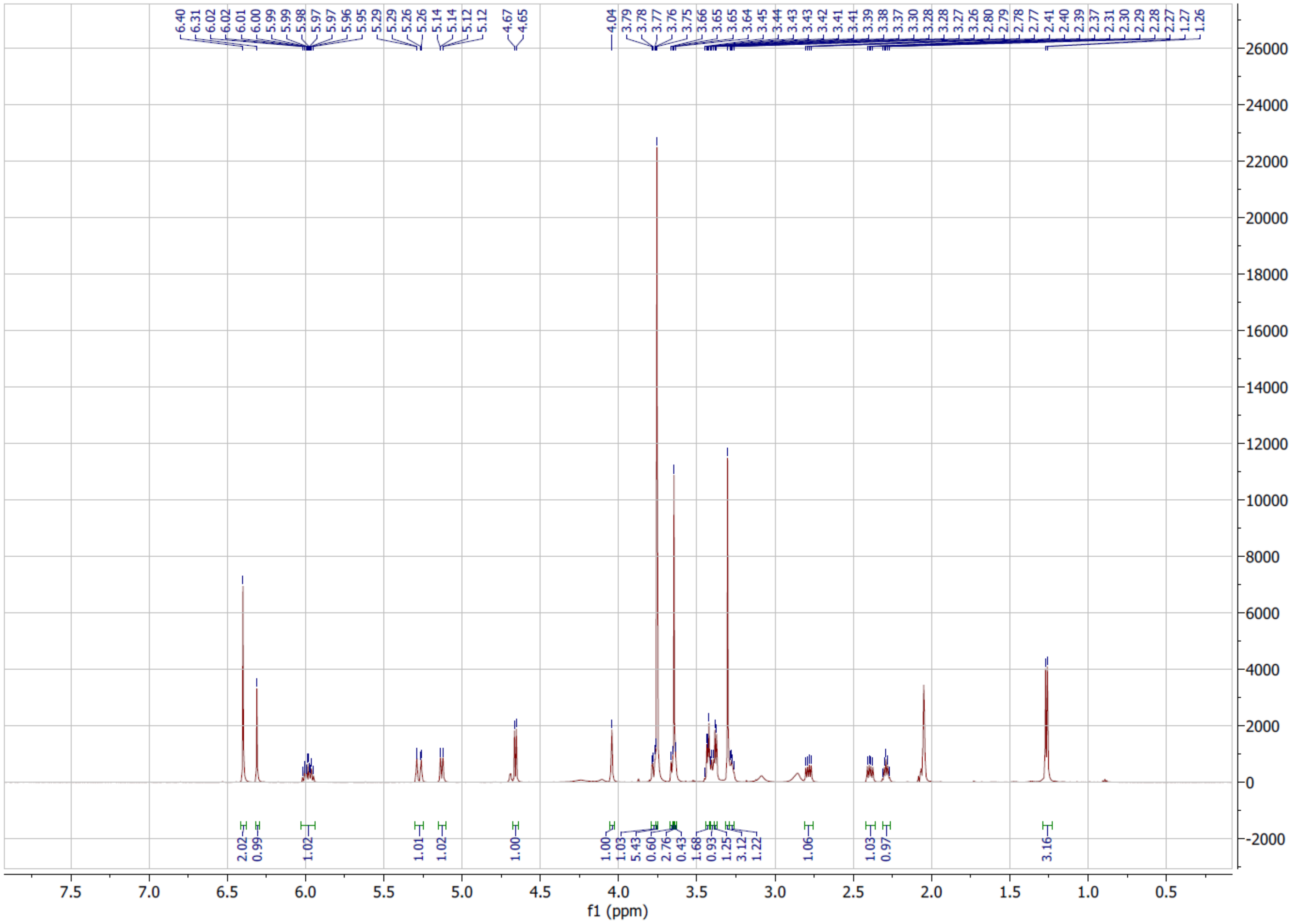

Figure S9. ^{1}H-NMR (600 MHz, acetone-d_6) spectrum of compound **2**

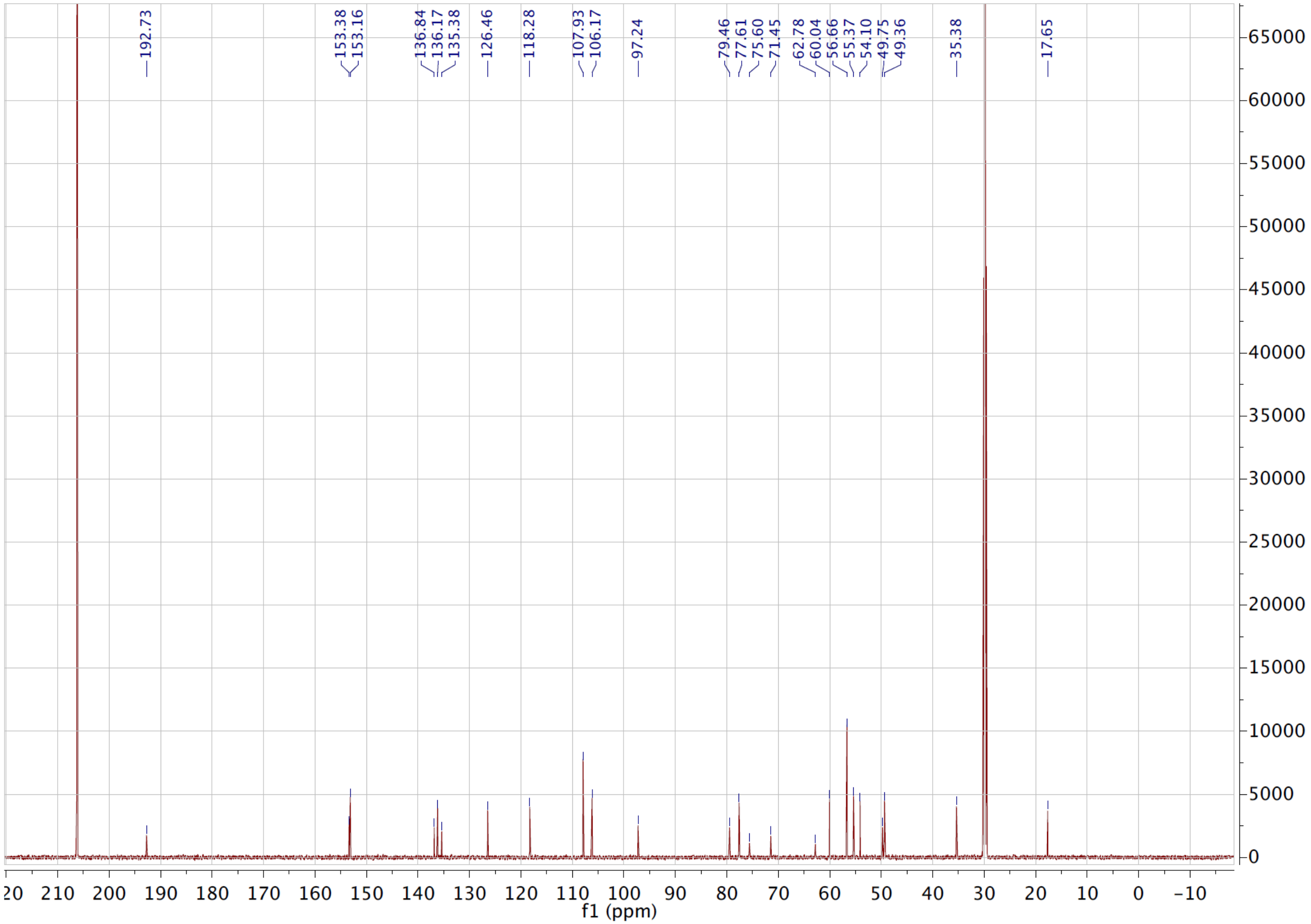

Figure S10. ^{13}C-NMR (150 MHz, acetone-d_6) spectrum of compound **2**

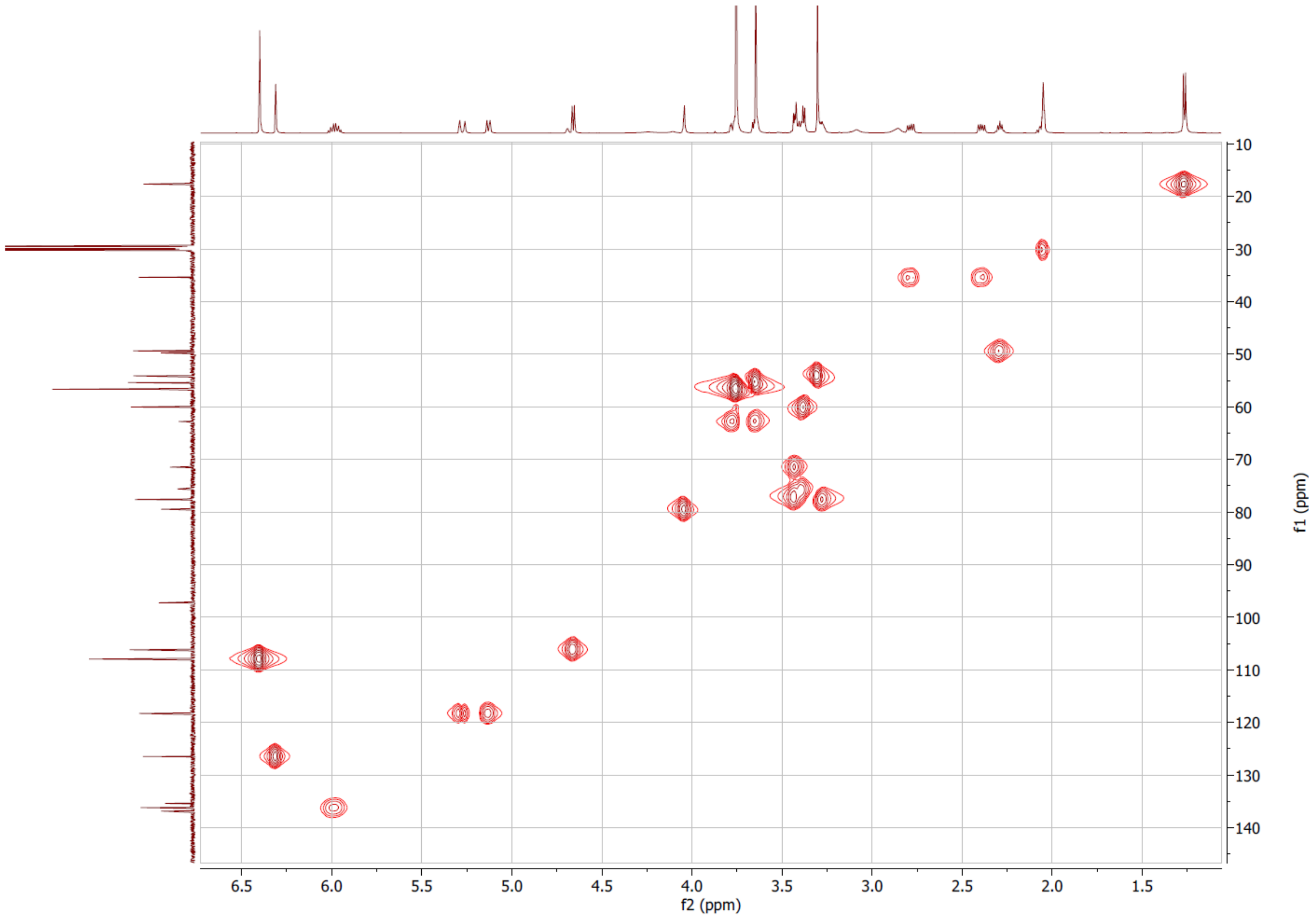

Figure S11. HSQC spectrum of compound **2**

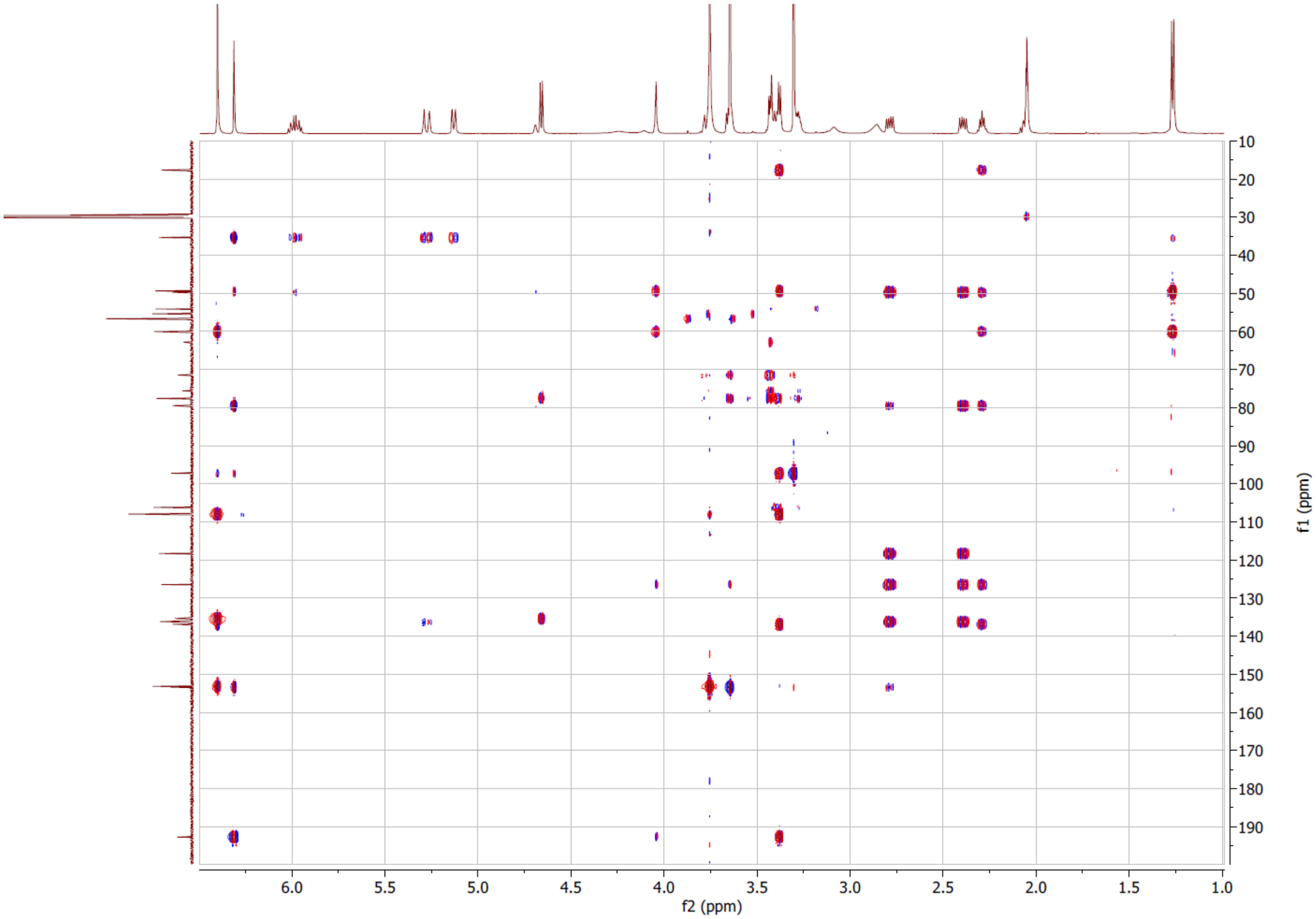

Figure S12. HMBC spectrum of compound

2 15

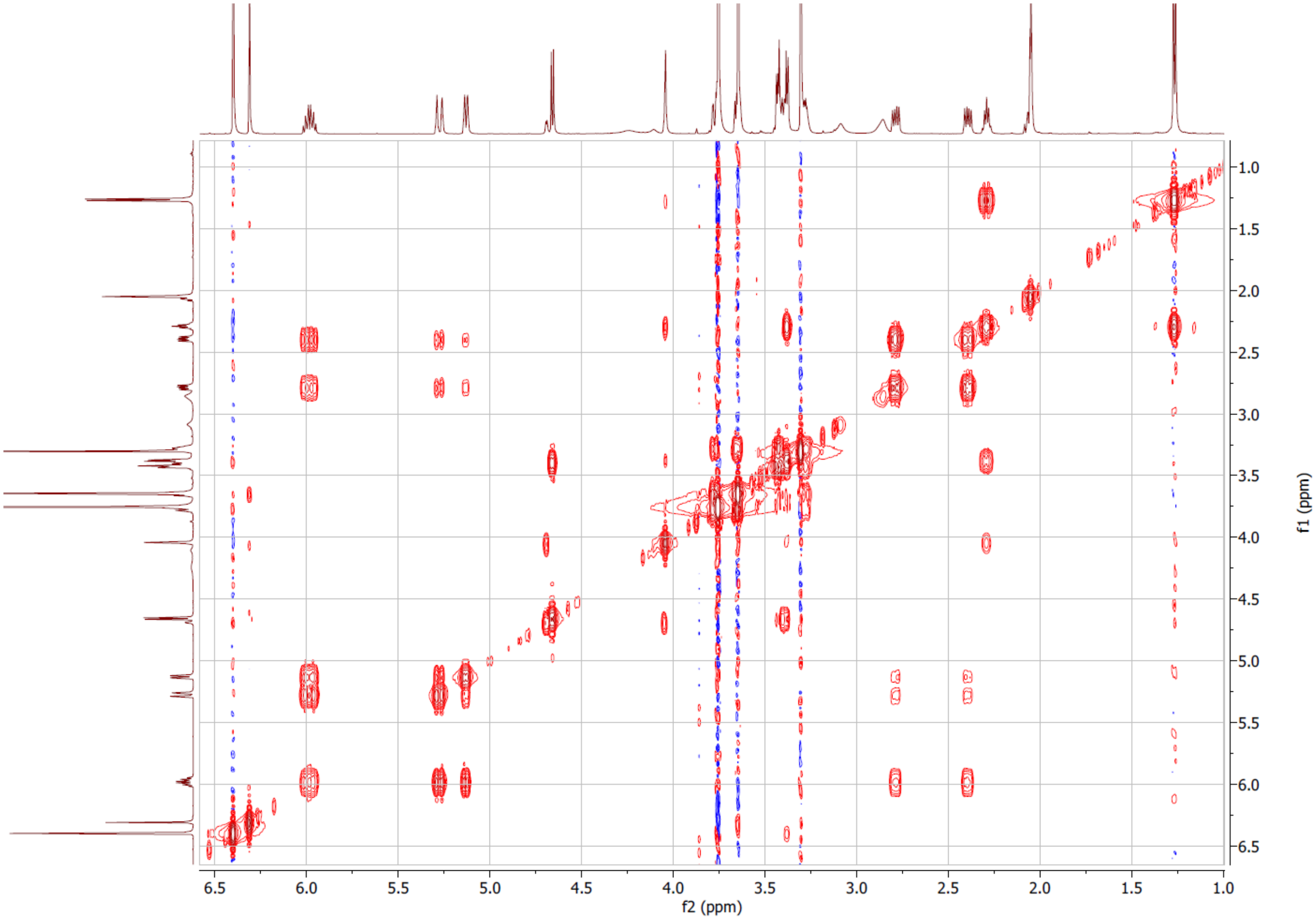

Figure S13. COSY spectrum of compound

2 16

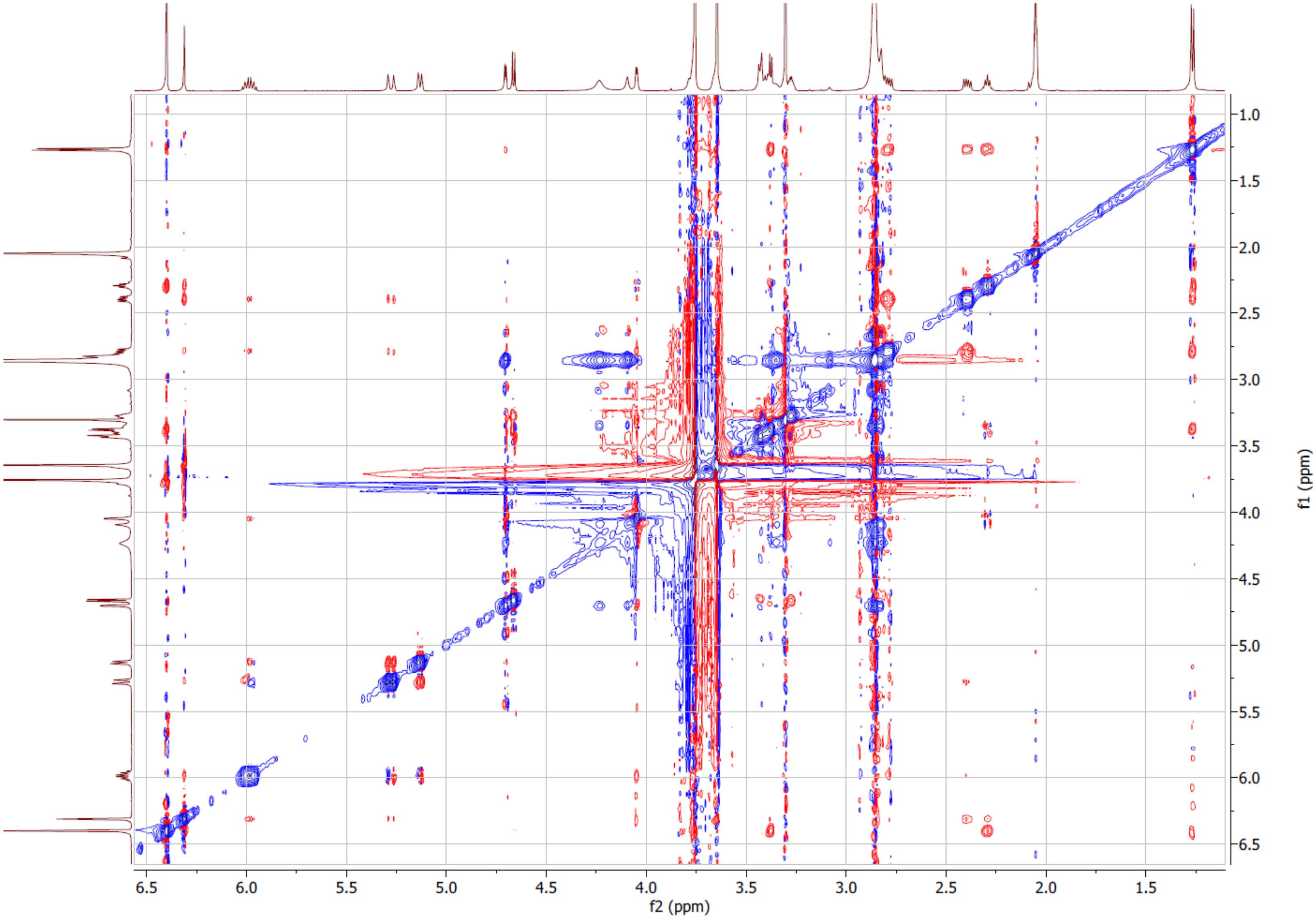

Figure S14. ROESY spectrum of compound

2 17

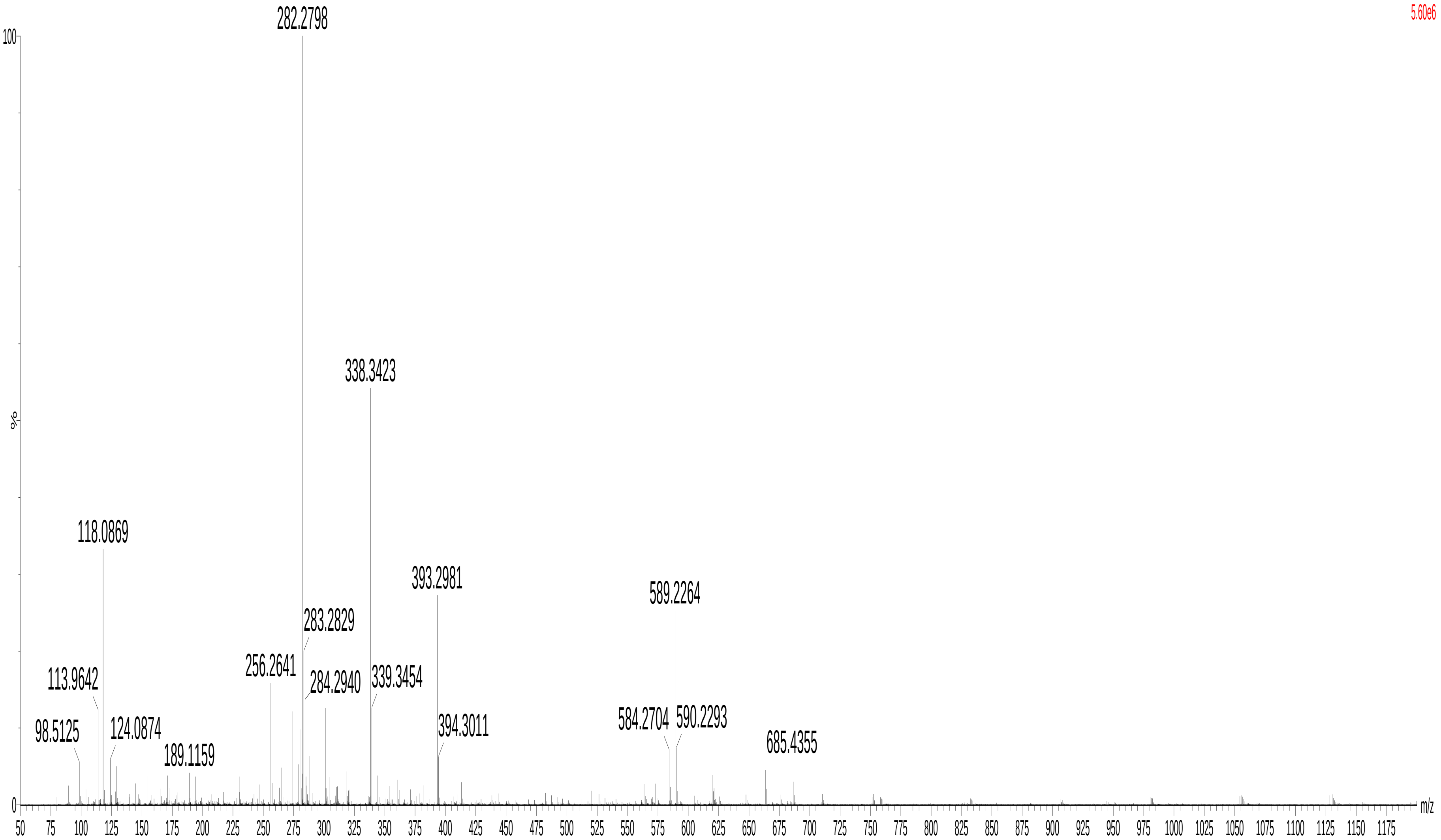

Figure S15. HR-ESI-MS spectrum of compound **2**

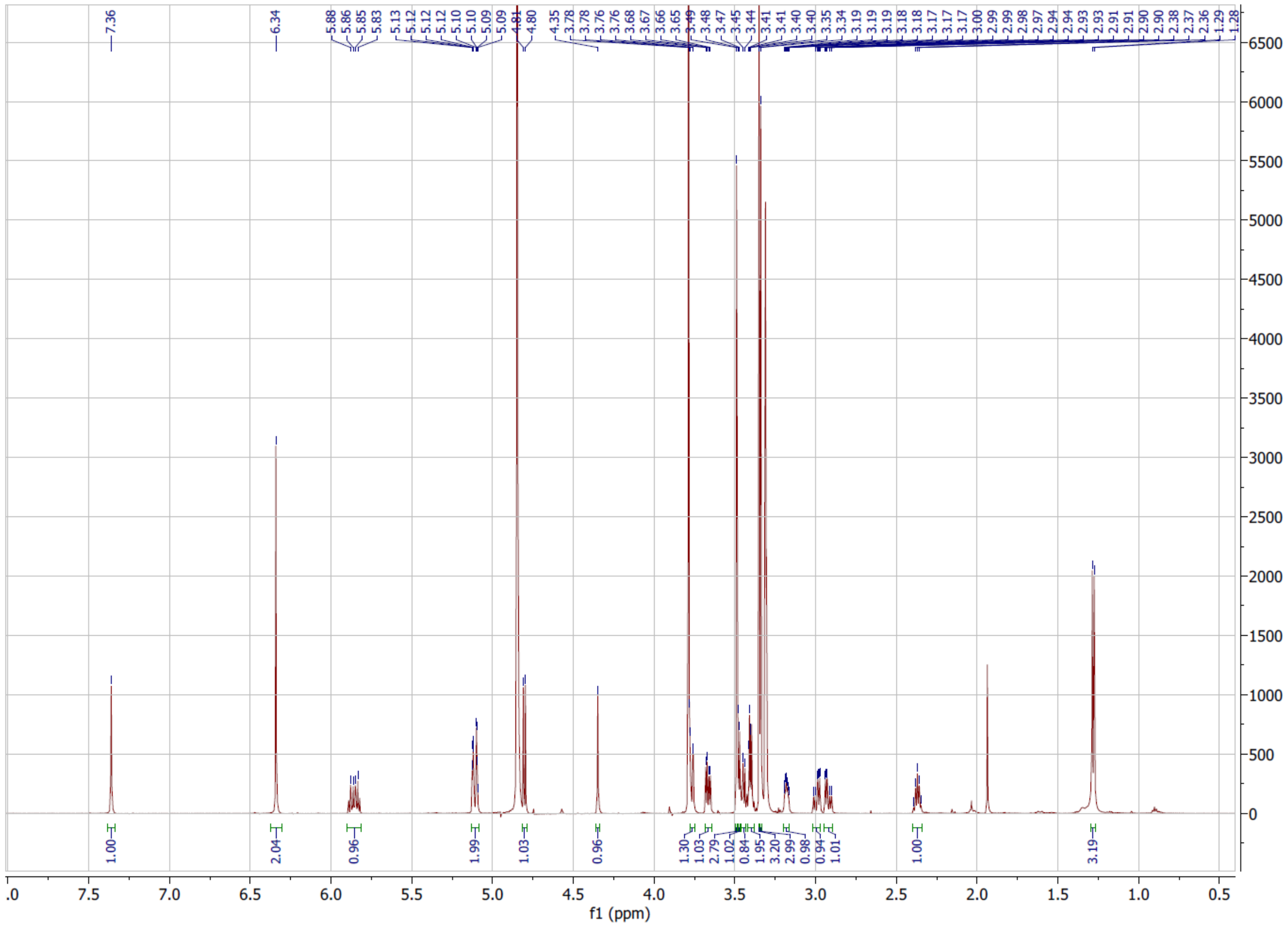

Figure S16. ^{1}H-NMR (600 MHz, methanol-d_4) spectrum of compound **3**

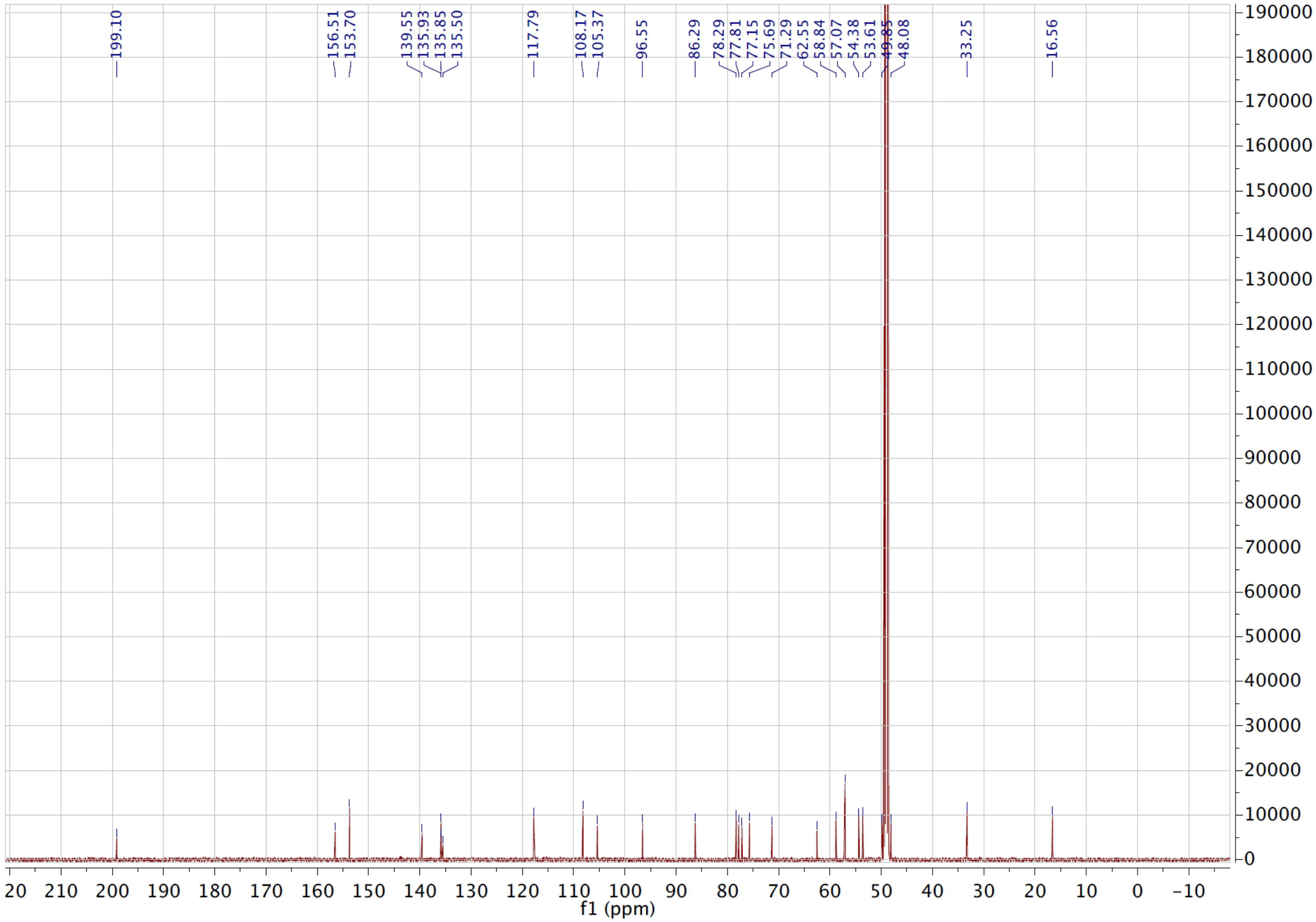

Figure S17. ^{13}C-NMR (150 MHz, methanol-d_4) spectrum of compound **3**

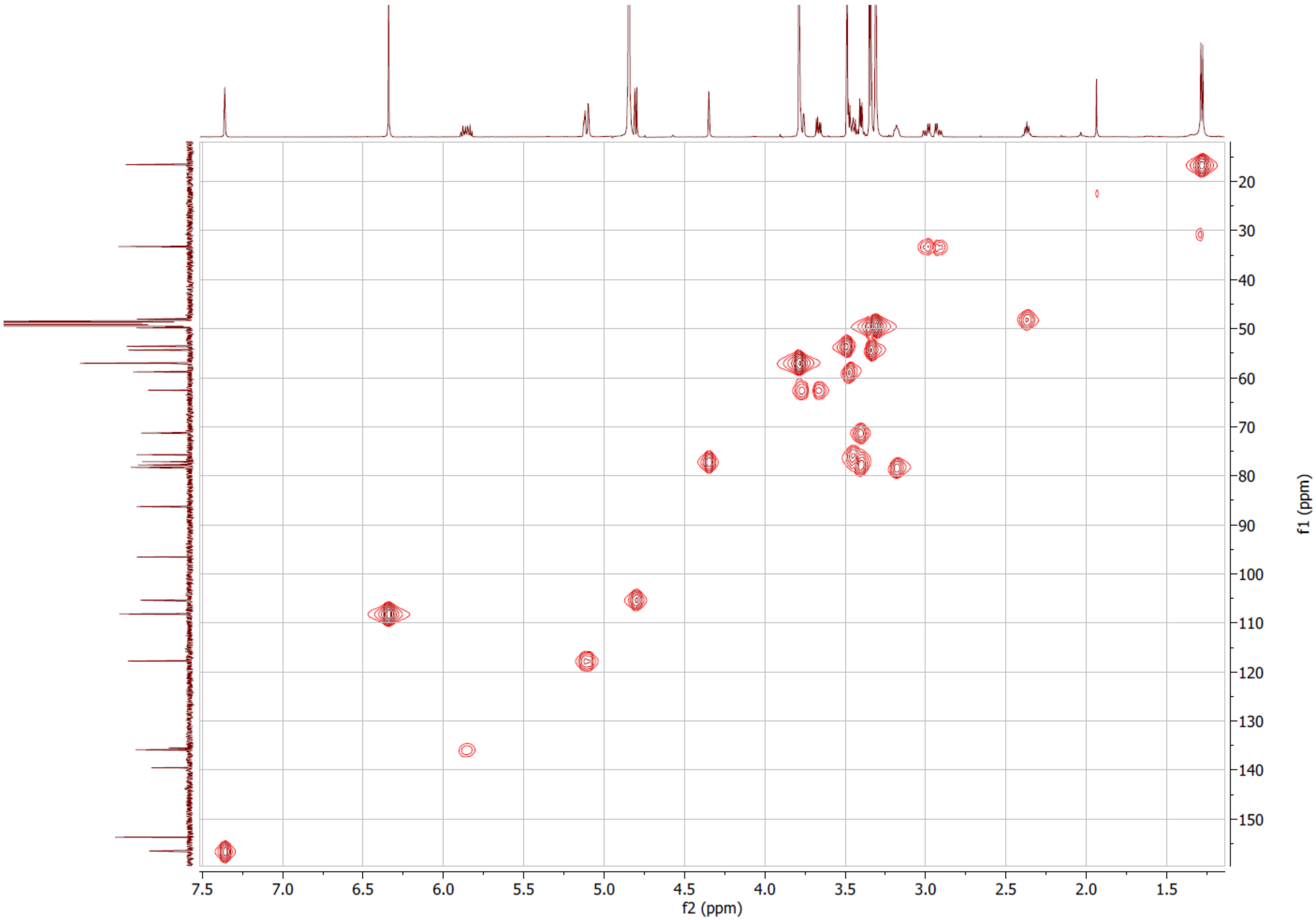

Figure S18. HSQC spectrum of compound

3 21

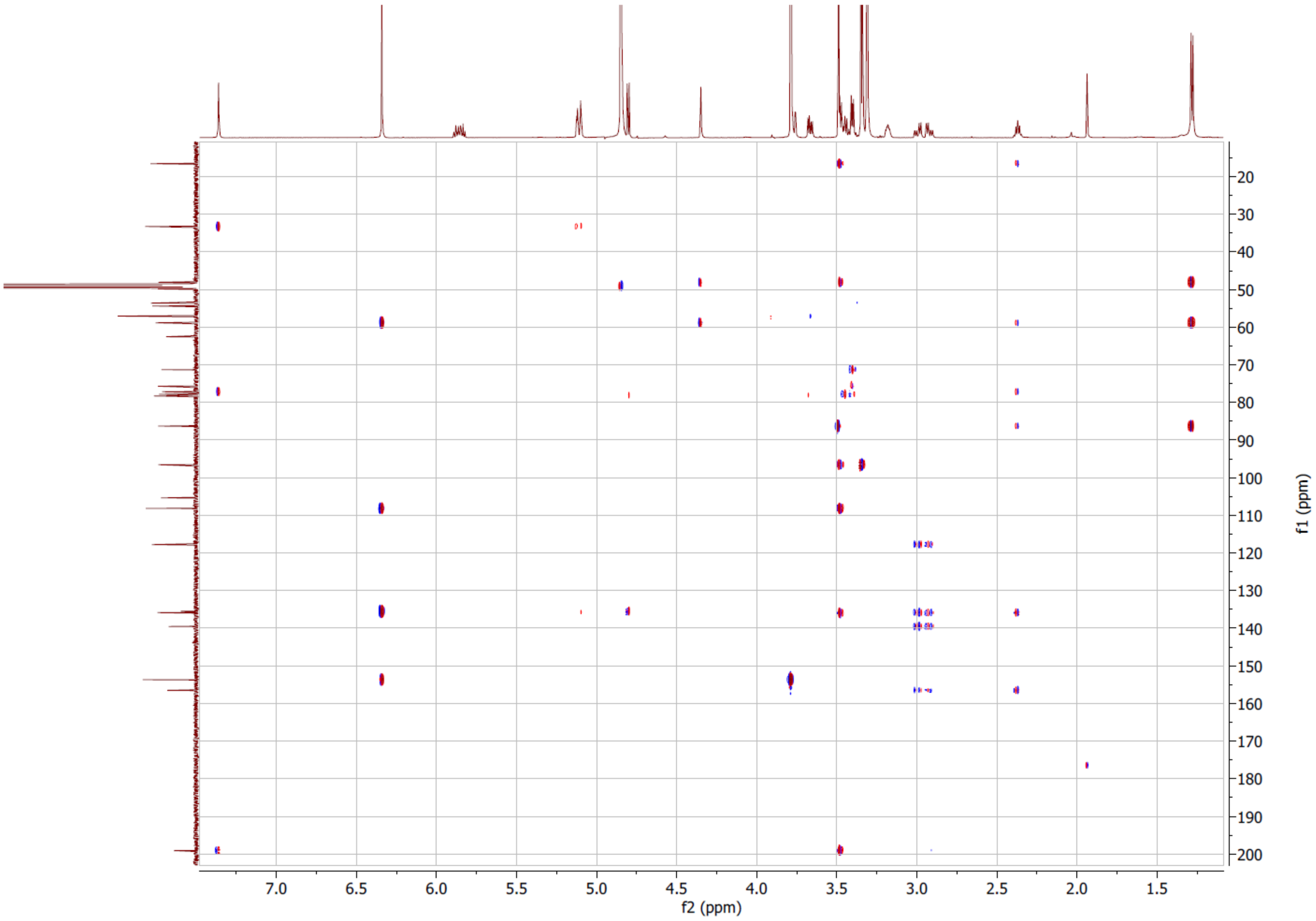

Figure S19. HMBC spectrum of compound

3 22

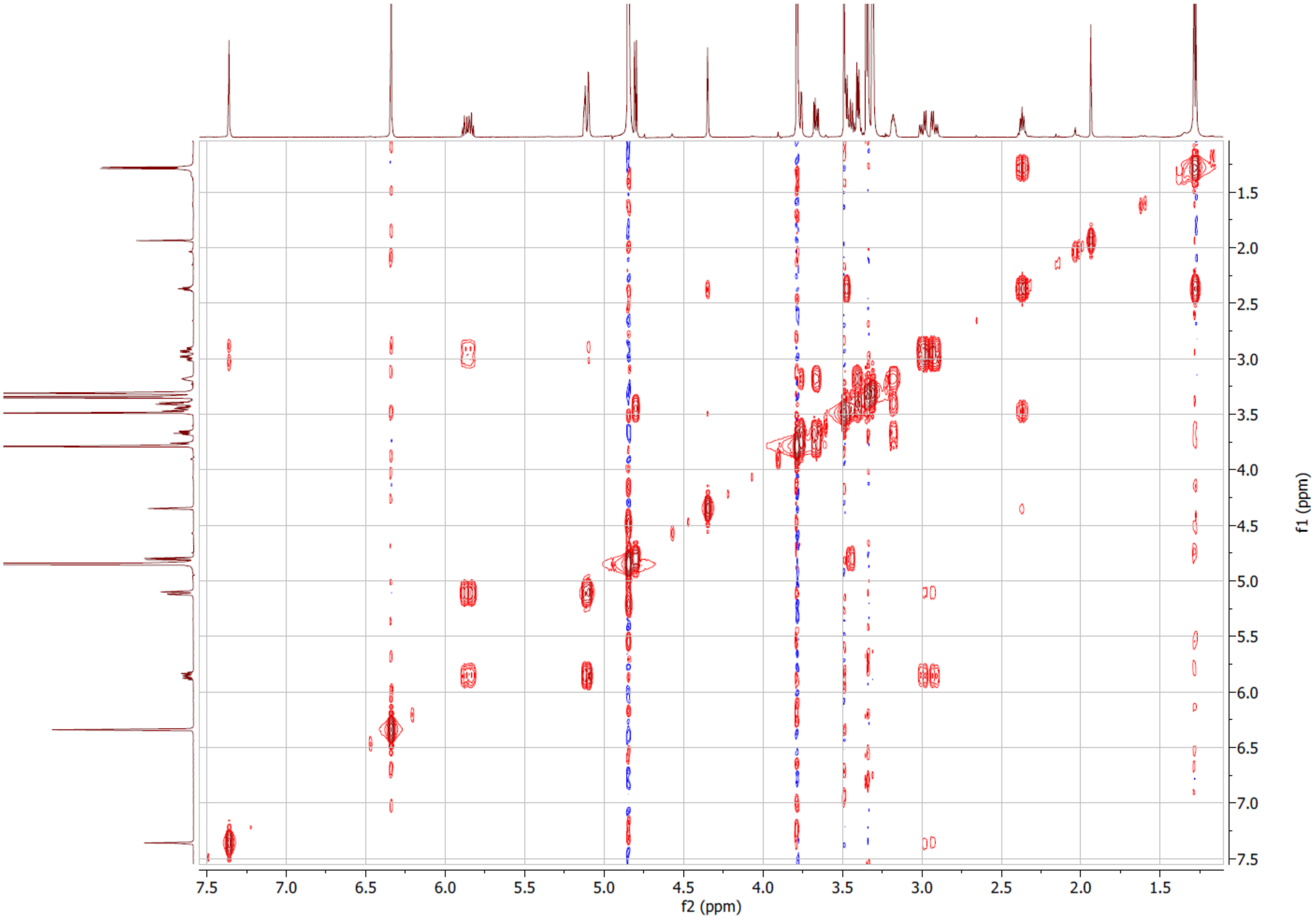

Figure S20. COSY spectrum of compound

3

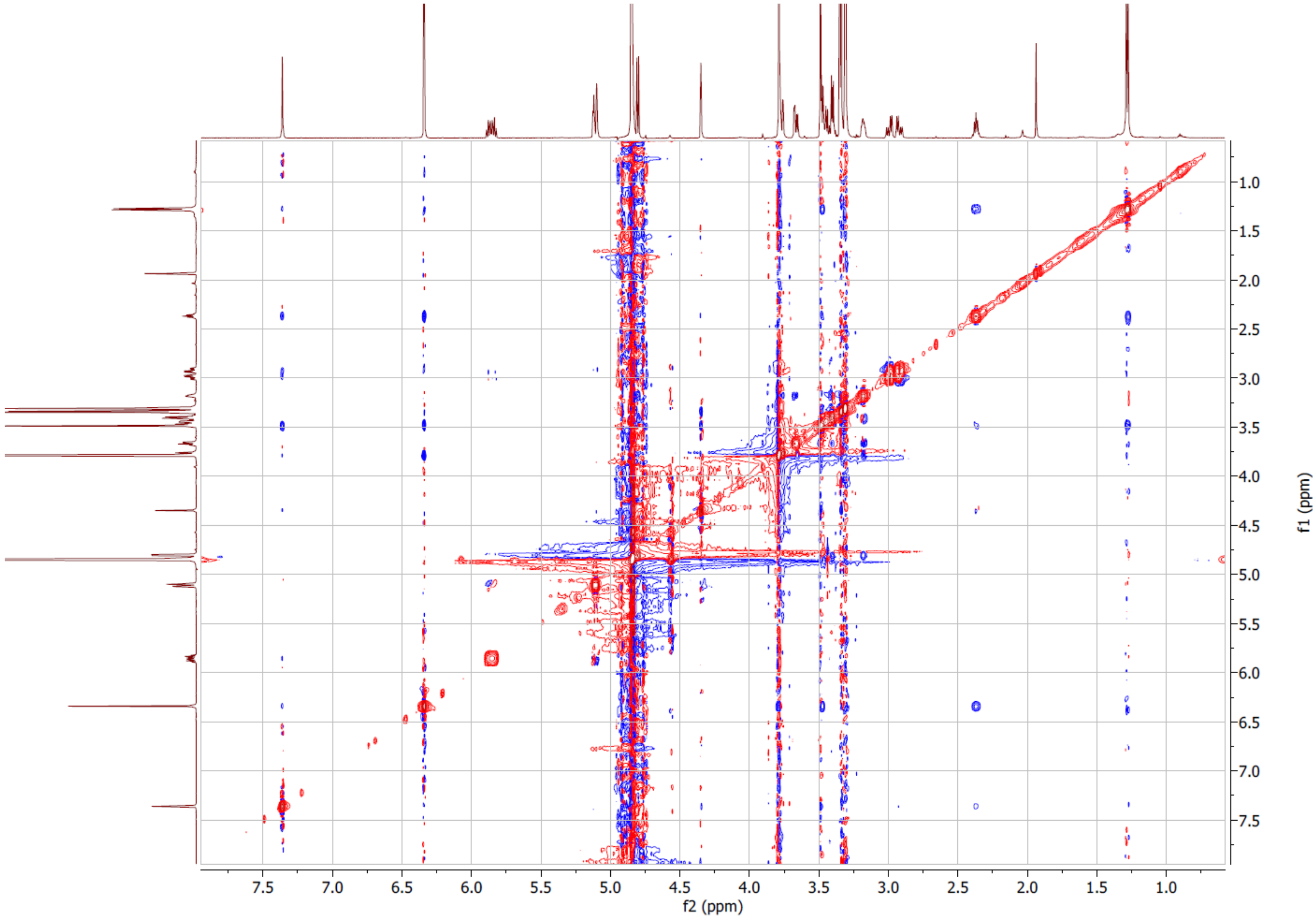

Figure S21. ROESY spectrum of compound

3

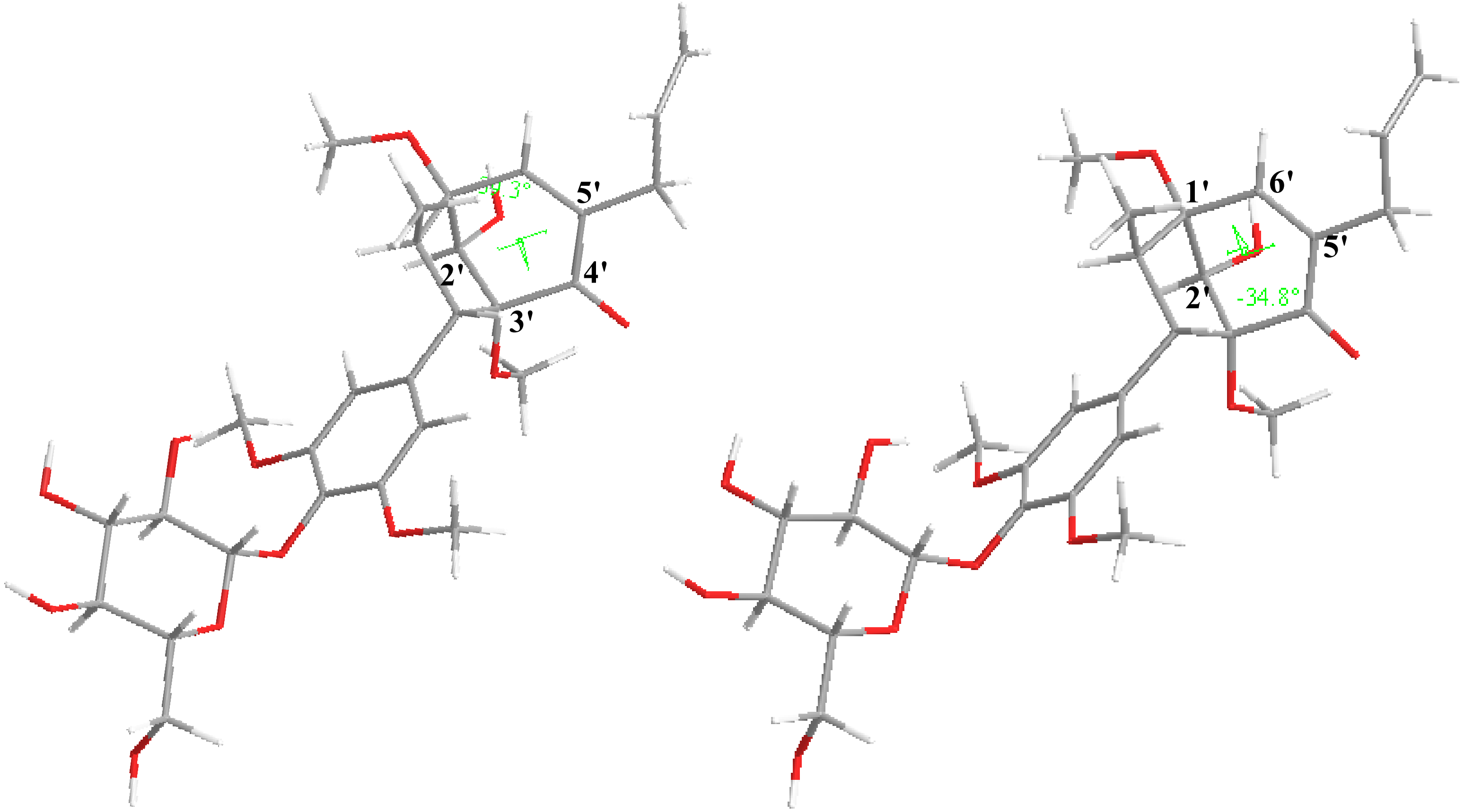

Figure S22. Adjacent torsion angles of compound

3

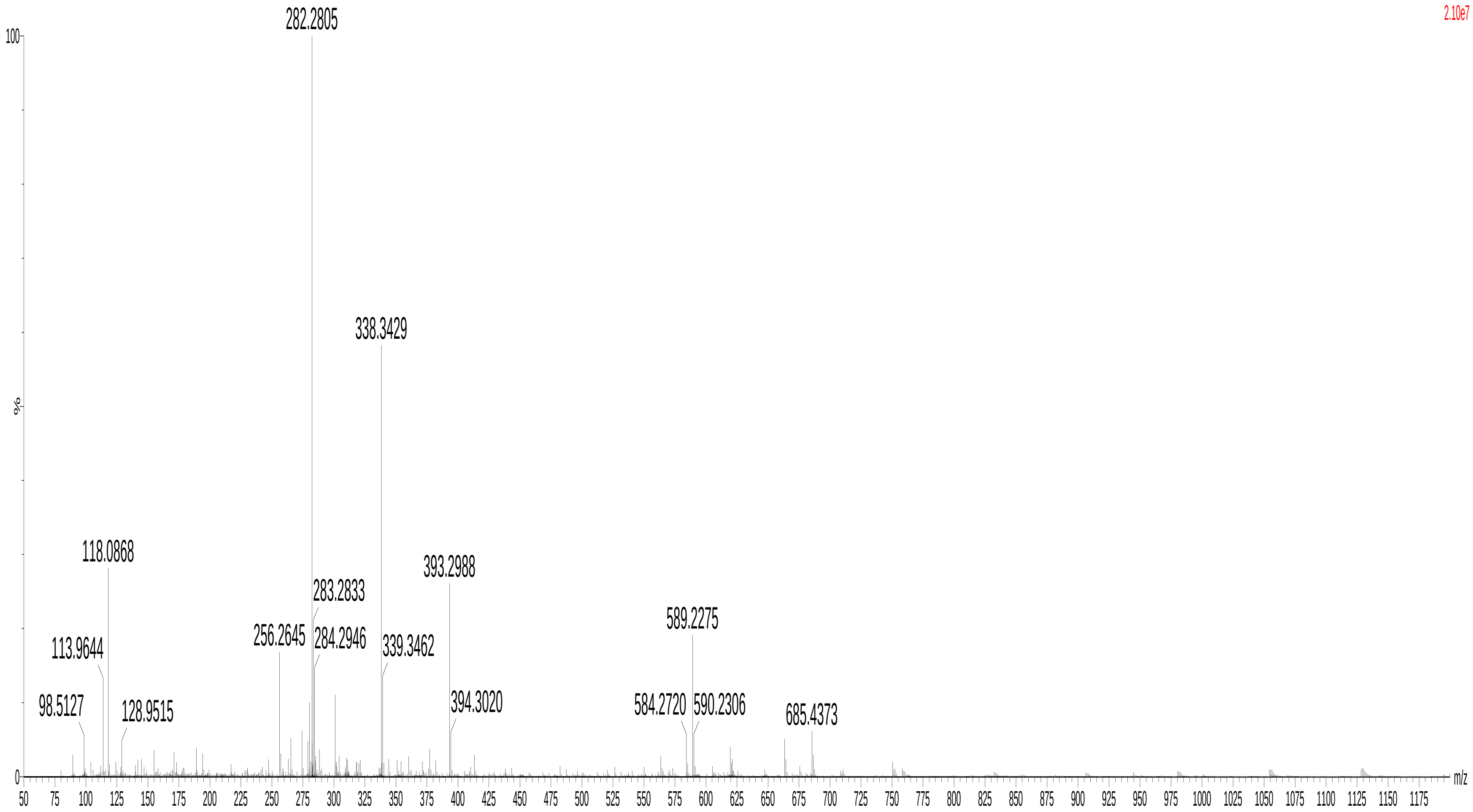

Figure S23. HR-ESI-MS spectrum of compound **3**

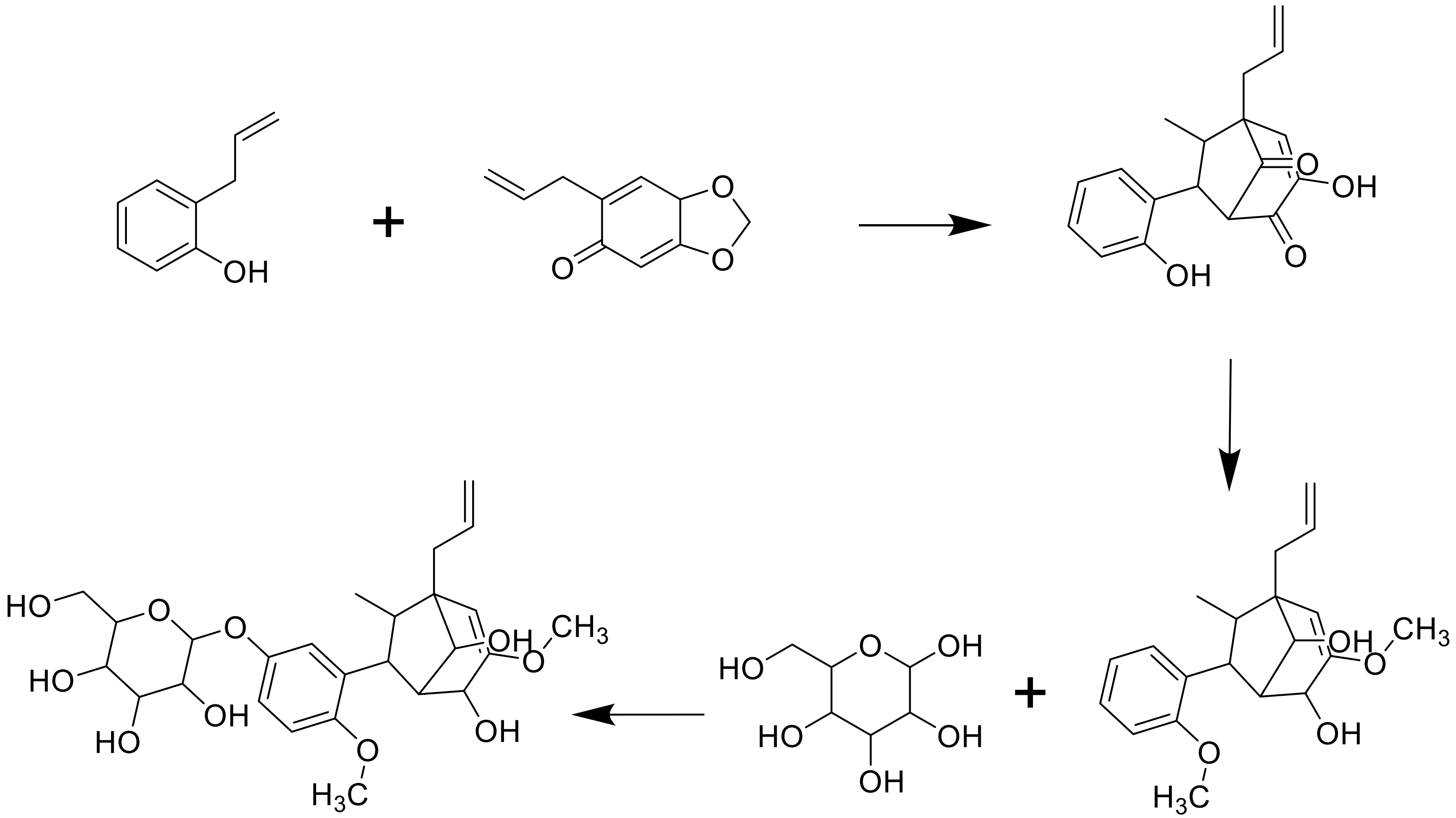

Figure S24. Speculated biosynthetic pathway of compound **1**

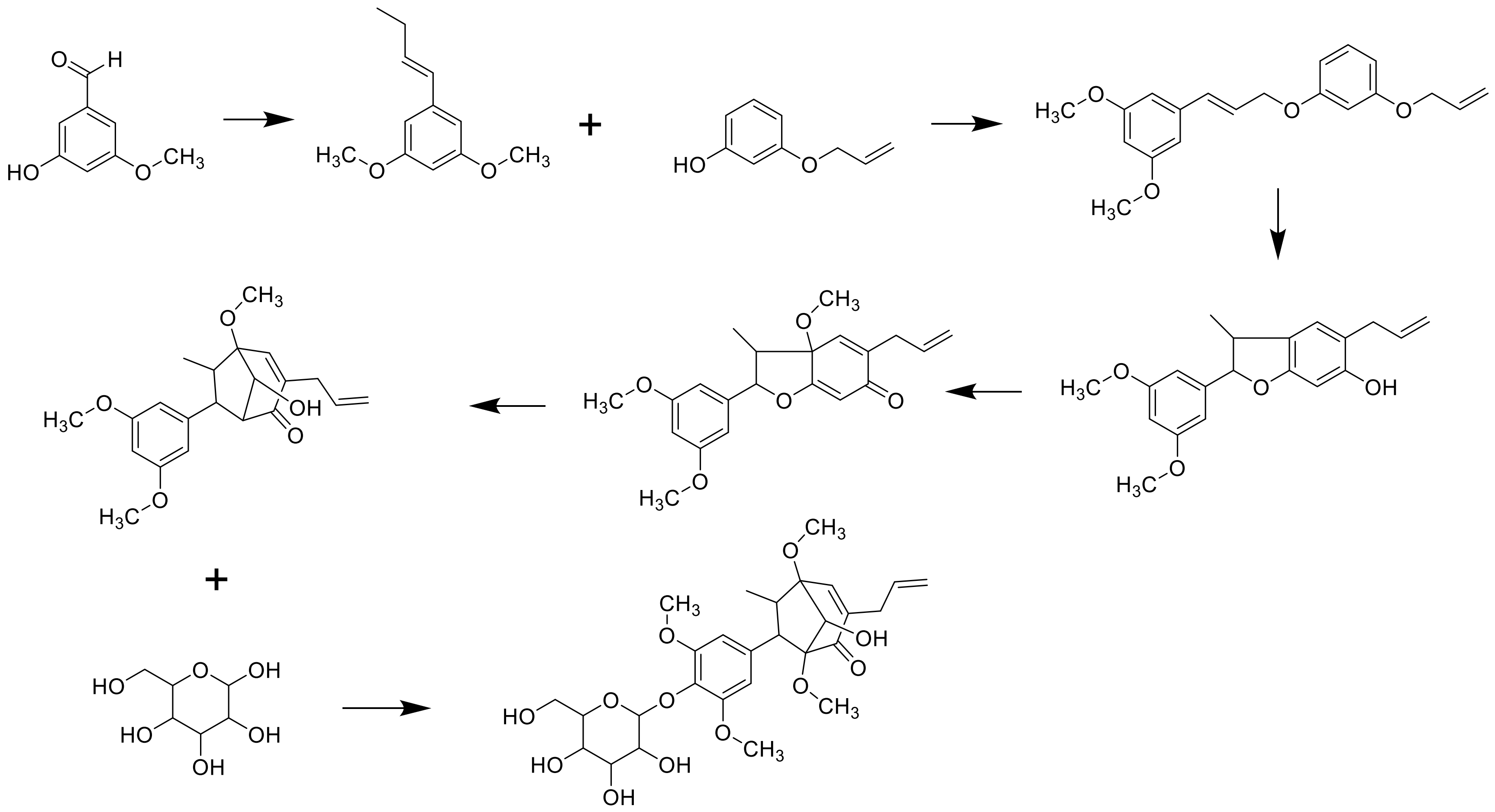

Figure S25. Speculated biosynthetic pathway of compound **3**
